# Identification of a heterologous cellulase and its N-terminus that can guide recombinant proteins out of *Escherichia coli*

**DOI:** 10.1186/s12934-015-0230-8

**Published:** 2015-04-10

**Authors:** Dongfang Gao, Shengjun Wang, Haoran Li, Huili Yu, Qingsheng Qi

**Affiliations:** State Key Laboratory of Microbial Technology, Shandong University, Jinan, 250100 People’s Republic of China

**Keywords:** *Escherichia coli*, Cellulase, Extracellular protein production, N-terminal sequence

## Abstract

**Background:**

The Gram-negative bacterium *Escherichia coli* has been widely used as a cell factory for the production of proteins and specialty chemicals because it is the best characterized host with many available expression and regulation systems. However, recombinant proteins produced in *Escherichia coli* are generally intracellular and often found in the form of inclusion bodies. Extracellular production of proteins is advantageous compared with intracellular production because extracellular proteins can be purified more easily and can avoid protease attack, which results in higher product quality. In this study, we found a catalytic domain of a cellulase (Cel-CD) and its N-terminus can be employed as carriers for extracellular production of recombinant proteins.

**Results:**

In this report, we identified the catalytic domain of a cellulase (Cel-CD) from *Bacillus* sp. that can be secreted into the medium from recombinant *E. coli* BL21 (DE3) in large quantities without its native signal peptide. By subcellular location analysis, we proved that the secretion was a two-step process and the N-terminal sequence of the full length Cel-CD played a crucial function in secretion. Both the Cel-CD and its N-terminal sequence can serve as carriers for efficient extracellular production of select target proteins.

**Conclusions:**

Fusion of heterologous proteins with N20 from Cel-CD can carry the target proteins out of the cells with a concentration from 101 to 691 mg/L in flask cultivation. The extracellular recombinant proteins with a relative high purity. The results suggested that this system has a potential application in plant biomass conversion and industrial production of enzymes and therapeutic proteins.

## Introduction

The Gram-negative bacterium *Escherichia coli* has been widely used as a cell factory for the production of enzymes and therapeutic proteins, because it is the best characterized host with many available expression and regulation tools [1-3]. However, the common laboratory strains of *E. coli* are poor secretors of proteins under normal culture conditions, because this bacterium has a complex cell envelope with two layers [4-6]. Therefore, heterologous proteins produced in recombinant *E. coli* are generally intracellular and often in the form of inclusion bodies, from which the biologically active proteins can only be recovered by complicated and costly processes [7]. Extracellular production of heterologous proteins in *E. coli* will not only provide a simple and convenient production and purification process, but also provide fast and direct screening capabilities for target therapeutic proteins or enzymes that are heterologously expressed in recombinant *E. coli* [8].

Significant effort to produce target proteins extracellularly in *E. coli* has been made, with the research efforts split into two categories. (1) Targeted accumulation of the heterologous protein in the periplasmic space through the inner membrane (IM) using a leader peptide, such as PelB, then the heterologous protein is released to the medium through the outer membrane (OM) using cell envelope mutants or lysis proteins [9-11]; (2) Fusion of the heterologous proteins to fusion partners that can be secreted from the cytosol out of the cells via known or unknown systems.

A number of signal peptides have been used for secretory production of recombinant proteins in both eukaryote and prokaryote [12,13]. The typical signal sequence most located in the amino terminal of proteins that functions as a targeting and recognition signal and contains a cleavage site which can be cleaved by a special signal peptidase after transportation [14]. However, the recombinant proteins fused with signal sequence are transported to the periplasmic space instead of culture medium due to the double membrane structure of *E. coli* [15]. To release the target proteins to the culture medium, cell envelope mutants or lysis proteins were employed. But it also suffers from the purity of the secreted proteins and cell sensitivity to the environment due to the leakage of the cell envelope.

Regarding the application of the second approach, several proteins, including heterologous proteins, were found to be secreted directly into the medium from recombinant *E. coli*. However, only a few of proteins were investigated as fusion partners for extracellular recombinant protein production, because the secretion efficiency and universality were not sufficient to scale to a larger expression volume [16-20]. In 2005, Majander *et al.*, modified the flagellar type III secretion apparatus of *E. coli*. Then, by fusing a heterologous protein between the 173-bp untranslated region upstream of the gene *fliC* (encoding flagellin) and a transcriptional terminator from *fliC*, they found that the target proteins can be secreted into the medium via the type III secretion apparatus at levels of 1 to 15 mg/L [21]. Subsequently, researchers identified a 13 kDa endogenous bacterial peptide in *E. coli*, YebF, which can be secreted into the medium in relatively large amounts by a two-step process via the periplasmic space [22]. Recently, an osmotically inducible protein Y (OsmY) from *E. coli* BL21 (DE3) was identified using a proteomic method. OsmY can also be secreted out of the cells with target proteins at concentrations ranging between 5 and 64 mg/L [20]. Furthermore, ESETEC®, WACKER’s patented secretion system, a technology for producing proteins and antibody fragments on a patented strain of *E. coli* K12, was the only one commercial solution to our knowledge [23].

Above all, it is generally considered that no fusion secretion systems are suitable for all heterologous proteins, because current fusion partner systems have been shown to have limitations [24-26]. In this study, we report that the catalytic domain of a cellulase (Cel-CD) from *Bacillus* sp*.*Z-16 can be efficiently secreted from *E. coli* when it was heterologously overexpressed*.* The accumulation of Cel-CD in the culture medium reached 514 mg/L. As the cellulase plays an important role in cellulosic biomass transformation, the extracellular expression of Cel-CD in *E. coli* provides a platform for cellulose production. Both the Cel-CD and its N-terminal sequence have potential as fusion partners in the production of various recombinant proteins.

## Results

### Cel-CD is secreted from *E. coli* into the medium without typical signal peptides

A previously screened *Bacillus* sp*.* Z-16 was found to produce alkaline cellulase with endo-beta-glycosidase activity. This enzyme is composed of a 30-residue signal peptide at N-terminus, a catalytic domain (GH5, residues 30 to 404), followed by two carbohydrate-binding domains at C-terminal region (CBM_17_28, residues 405 to 821). The catalytic domain of this enzyme (Cel-CD) is 100% identical with a cellulase from *Bacillus subtilis* in the NCBI (DNA similarity is 99%, accession No.: M84963, 1,494 to 2,618). The full length Cel-CD consists of 375 amino acids with a molecular mass of 41.53 kDa.

**Figure 1:**
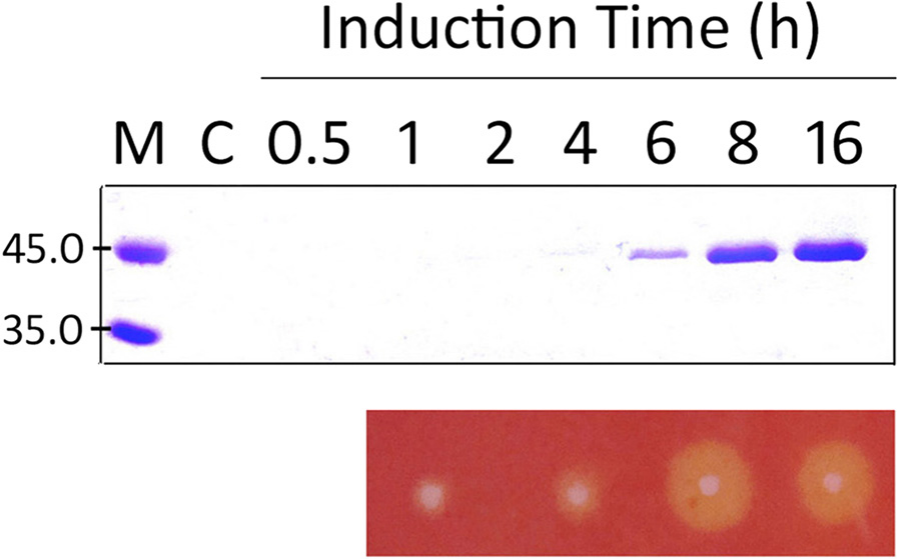
**Secretion analysis of Cel-CD in** ***E. coli*** **BL21 (DE3).** SDS-PAGE analysis and activity assay of the Cel-CD in the culture medium. Samples were collected by centrifugation at the indicated times after induction. The endoglucanase activity of the secreted Cel-CD was determined by Congo red staining as described in the Materials and Methods. Molecular size markers are shown in kDa. Lane M is the marker; Lane C is the control of collected cells.

We cloned the 1,125-bp coding region of the *cel-cd* gene from *Bacillus* sp*.* Z-16, without its upstream native signal sequence into the T7-driven expression plasmid pET28a, resulting in pET28a/cel. This recombinant plasmid was transformed into *E. coli* BL21 (DE3) cells for overexpression. During cultivation in the shake flask, we were surprised to find the Cel-CD in the culture medium not only in the cytosol. The Cel-CD progressively accumulated in the medium as the culture grew (Figure 1). After 24 h cultivation at 37°C, the final extracellular protein concentration reached 514 mg/L and the hydrolytic activity of Cel-CD towards carboxymethylcellulose (CMC) reached 558.4 U/L. Compare with the cells harboring pET28a, the overexpression of Cel-CD had no effect on the growth of *E. coli*.

Analysis of the N-terminal sequence of purified extracellular Cel-CD showed that it was MEGNTREDNF, which is the same as the predicted N-terminal sequence of the Cel-CD. The result proved that Cel-CD was translocated from *E. coli* BL21 (DE3) without removing any N-terminal sequence.

### The secretion of Cel-CD is a two-step process via the periplasmic space

**Figure 2:**
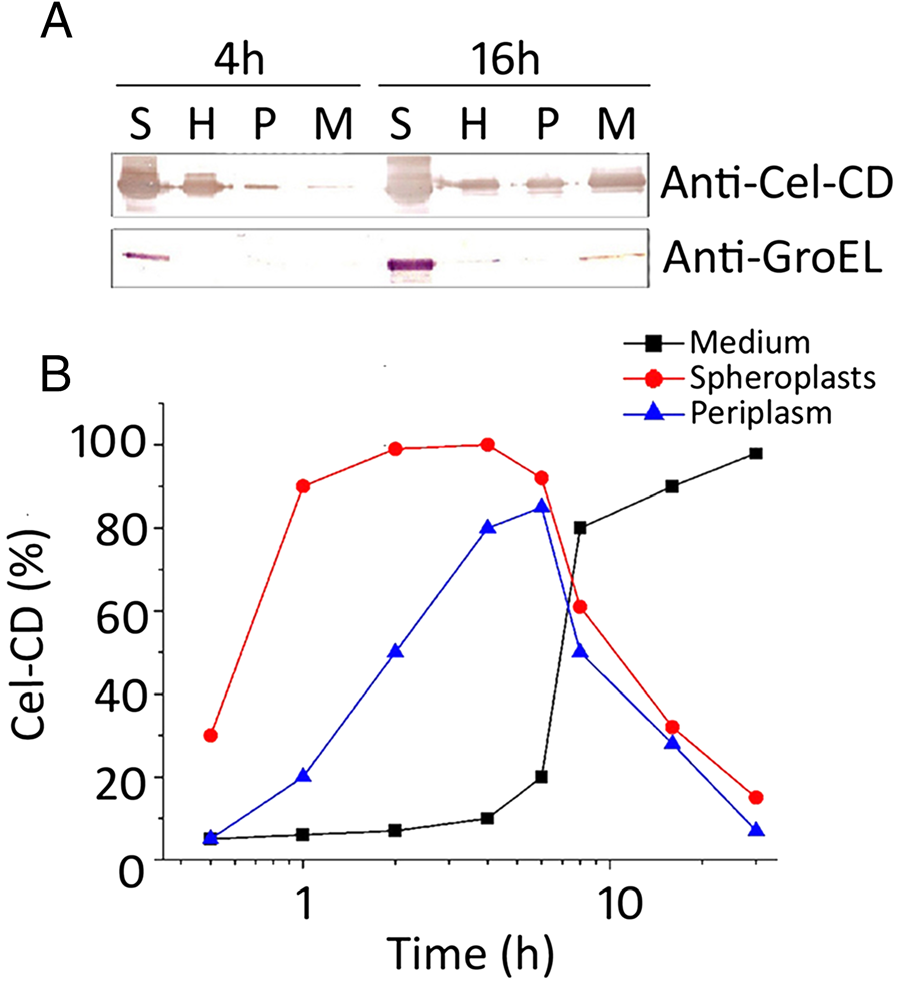
**Subcellular localization and accumulation process analysis of Cel-CD. (A)** Subcellular localization of Cel-CD. Cells were harvested 4 and 16 h after induction and washed with 1 mL of 100 mM MOPS (pH 7.0), then subjected to cold osmotic shock to separate the periplasm from spheroplasts. **(B)** The accumulation process of Cel-CD at different locations*.* Cells were harvested after 8 h induction and washed with 1 mL of 100 mM MOPS (pH 7.0), then suspended in 50 mL of fresh LB containing 80 μg/mL chloramphenicol. The quantification of Cel-CD was performed by Bio-Rad Quantity One Version 4.2.1. Lanes are marked as: S, spheroplasts; P, periplasm; H, hypertonic solution; M, medium.

The lack of signal peptide in Cel-CD prompted us to determine its subcellular location. The *E. coli* BL21 (DE3) harboring Cel-CD was analysised using the cold osmotic shock method, as described in the Materials and Methods. We found that Cel-CD was in the cytoplasm, periplasmic space and medium, which proved that the secretion of Cel-CD was through the periplasmic space by western blot analysis with anti-Cel-CD antibodies (Figure 2A). In a subsequent experiment, we added chloramphenicol in the culture to inhibit nascent protein biosynthesis [27], and observed that the quantity of Cel-CD in the periplasmic space exhibited an initial increase and subsequently decreased in quantity, whereas it progressively accumulated in the medium (Figure 2B). These findings show that the secretion of Cel-CD is a two-step secretion process via the periplasmic space.

### Cell lysis determination

**Figure 3:**
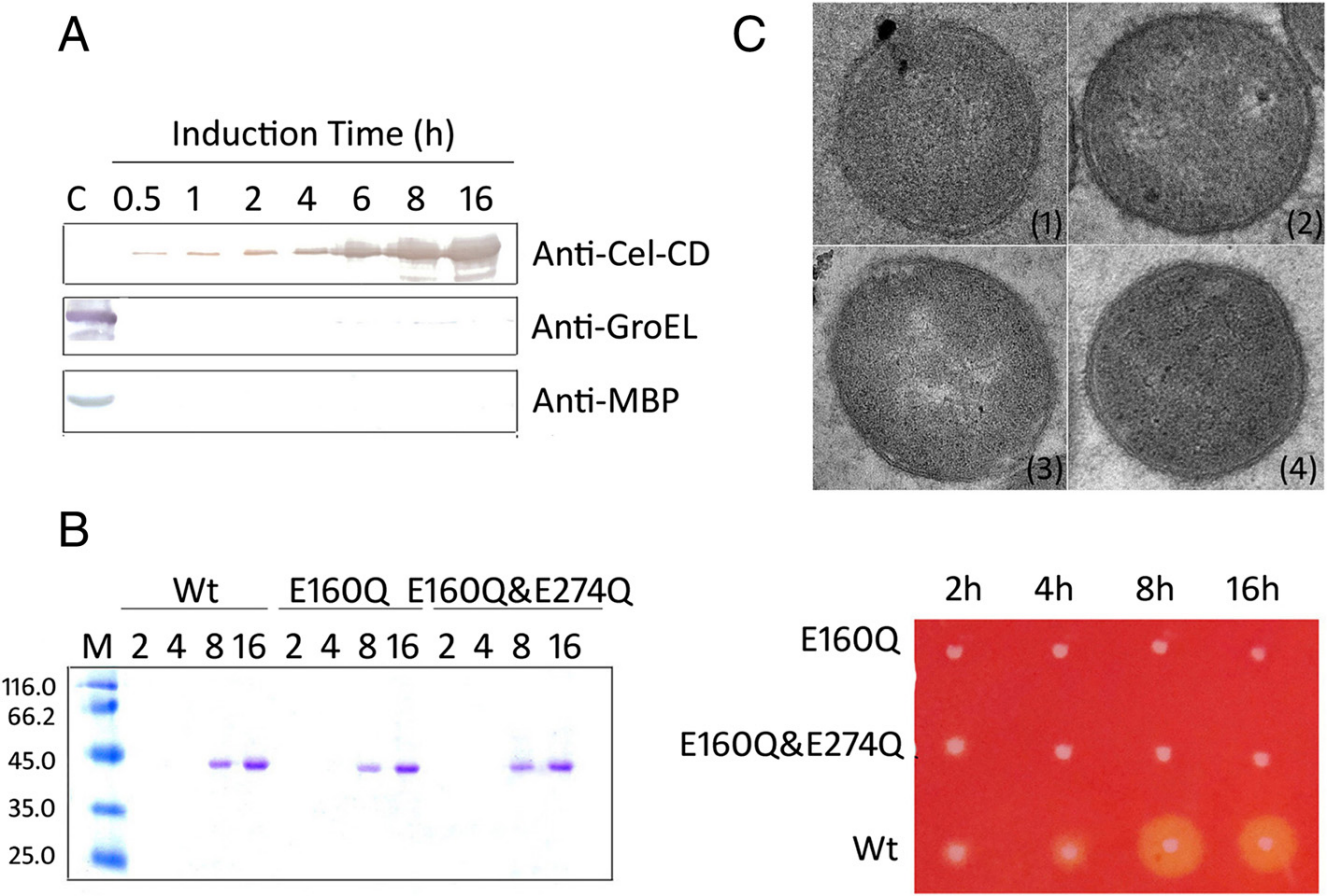
**Cell lysis determination and inactive Cel-CD secretion analysis. (A)** Immunoblotting detection of Cel-CD, GroEL and MBP in the culture medium. Samples were subjected to immunoblotting with an anti-Cel-CD antibody, an anti-GroEL antibody or an anti-MBP antibody. **(B)** SDS analysis of cellulase (wt) and its mutants (E160Q, E160Q&E274Q) secreted into the culture medium. The activities of the secreted mutants of Cel-CD (E160Q, E160Q&E274Q) were analyzed by Congo red staining method (4 h, 6 h, 8 h, 16 h). **(C)** TEM images of *E. coli* BL21 (DE3) that containing different plasmids. (1) pET28a/cel, (2) pET28a/E160Q, (3) pET28a/E160Q&E274Q, (4) pET28a. Protein samples were separated on 12% SDS-PAGE. Aliquots corresponding to 20 μL of the culture medium were loaded onto the gel. Molecular size markers are shown in kDa. Lane M is the marker; Lane C is the control of collected cells.

To exclude the possibility that extracellular Cel-CD accumulation was due to cell lysis or cell leakage through the outer membrane, we did western blot analysis against Cel-CD together with control proteins, the periplasmic maltose binding protein (MBP) [28] and the cytoplasmic protein GroEL [29]. The presence of control proteins in the medium would be an indication of cell lysis. As shown in Figure 3A, Cel-CD was detected in the culture medium at every time point, whereas MBP was not detected and GroEL was only detected in trace amounts after extended cultivation. This indicated that extracellular Cel-CD was not derived from cell lysis.

A recent report showed that the heterologously expressed cutinase from *Thermobifida fusca* was secreted from cells because of its hydrolytic activity toward phospholipids [30]. To exclude the possibility that Cel-CD secretion was also caused by its hydrolytic activity in the periplasmic space, the active site of Cel-CD was mutated according to the results of partial amino acid sequence alignment with cellulase. Two mutants, E160Q, and E160Q and E274Q, in which hydrolytic activity was completely lost, were still detected in the medium (Figure 3B). The morphology of *E. coli* BL21 (DE3) strains were analysised by Transmission electron microscopy (TEM). It showed that the cells expressing either Cel-CD or inactivated Cel-CD remained intact, supporting the aforementioned result (Figure 3C).

### The N-terminus of Cel-CD plays a key role in secretion

**Figure 4:**
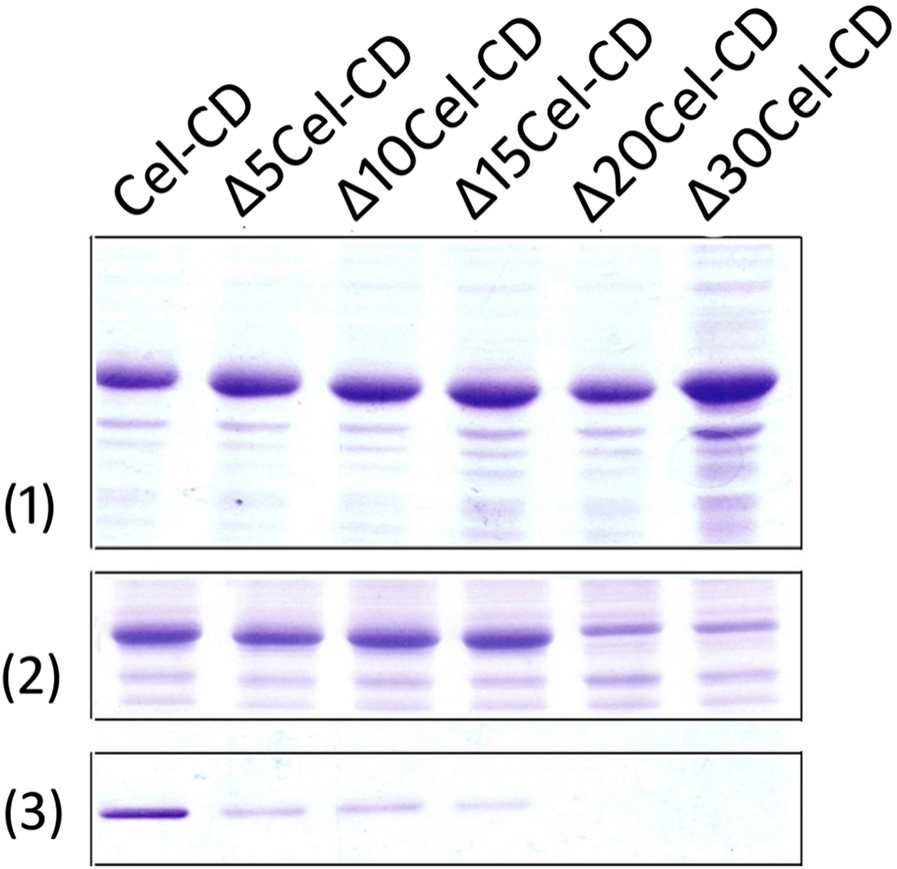
**The expression and secretion of Cel-CD and its N-terminal nested-deletion mutants.** Cells were harvested 16 h after induction and washed with 1 mL of 100 mM MOPS (pH 7.0). The whole cells **(1)**, the supernatant of the disrupted cells **(2)** and the released protein in the medium **(3)** were separated on 12% SDS-PAGE.

As the secretion of Cel-CD in *E. coli* BL21 (DE3) was a two-step pocess without predicted signal peptide, we further detected if the N-terminus of mature Cel-CD functioned as a signal peptide. We constructed five protein mutants (∆5Cel-CD, ∆10Cel-CD, ∆15Cel-CD, ∆20Cel-CD and ∆30Cel-CD) by gradual N-terminal truncation. The expression of the five mutants showed almost no difference, but the secretion level gradually decreased as the size of the truncation increased compared to the wild-type (Figure 4). This data demonstrate that the importance of the N-terminal sequence on Cel-CD secretion in *E. coli*. Moreover, the ∆20Cel-CD and ∆30Cel-CD mutants, which lack the N-terminal 20 or 30 residues, were completely inhibited the secretion. Taken together, these results indicated that the N-terminal 20 residues of Cel-CD may be the mini length for the export pocess.

### Extracellular production of heterologous proteins in *E. coli*

**Figure 5:**
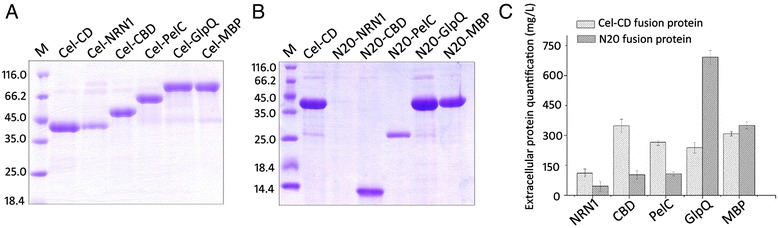
**Secretion analysis of the fusion proteins using Cel-CD/N20 as a partner. (A)** Fusion of target proteins with Cel-CD. Samples were collected after 24 h induction and separated on 12% SDS-PAGE. **(B)** Fusion of the target proteins with the N-terminal 20 residues of Cel-CD (N20). Samples were collected after 24 h induction and separated on 15% SDS-PAGE. **(C)** Quantitative comparison of extracellular proteins using different fusions. The dark gray column represents the Cel-CD fusion, whereas the light grey column represents the N20 fusion. Aliquots corresponding to 20 μL of the culture medium were loaded onto the gel. Molecular size markers are shown in kDa. The quantification of Cel-CD was performed by Bio-Rad Quantity One Version 4.2.1.

The efficient and quantitative secretion of Cel-CD inspired us to test if it could be used as a fusion partner or signal peptide/protein to carry other proteins out of the cell in recombinant *E. coli.* We selected a series of proteins with different sizes and sources, including pectate lyase C (PelC, 24.3 kDa) from *B. subtilis*, human neuritin (NRN1, 15.3 kDa), the carbohydrate binding domain of cyclodextrin glycosyltransferase (CBD, 11.21 kDa) from *B. circulans*, maltose-binding protein (MBP, 43.4 kDa) and glycerophosphoryl diester phosphodiesterase (GlpQ, 40.8 kDa) from *E. coli.* These five proteins can only be detected in the cytoplasm when they were expressed without any signal sequences. When fused downstream of Cel-CD, all these proteins were detected in the medium after 24 h cultivation; however, the secretion levels varied (Figure 5A). The secretion of Cel-CBD was the highest, reaching 348 mg/L. The secretion of Cel-glpQ, Cel-MBP, Cel-pelC and Cel-NRN1 in the culture medium was also substantial with values of 266.8, 307.6, 264.6 and 211.3 mg/L, respectively.

Since the N-terminal region plays an important role in secretion, we tried the N-terminal 20 residues (N20) as a signal peptide to guide the target proteins out of *E. coli* (Figure 5B). The N-terminal 20 residues from Cel-CD were added to the target proteins by direct PCR ligation. Fusion of N20 with GlpQ and MBP led to a significant increase in secretion when compared with the Cel-CD fusion (Figure 5C). The extracellular protein was 691 and 350 mg/L, respectively, while the secretion of N20-CBD and N20-pelC reduced to 101 and 106.7 mg/L, respectively. However, N20-NRN1 was not secreted. Analysis of the expression of N20-NRN1 showed that it was deposited as inclusion bodies (data not shown).

## Discussion

In this study, we have shown that Cel-CD, the catalytic domain of a cellulase from *Bacillus* sp.Z-16, could be secreted into the culture medium without a typical signal sequence when expressed in *E. coli* BL21 (DE3)*.* As a heterologous protein, the overexpression of Cel-CD was not found any interference to cell growth and metabolism. This may overcome the cell lysis caused by the overexpression of the fusion partner, thus extend the fermentation process to increase the extracellular production. Meanwhile, the secretion of Cel-CD and its recombinant proteins was a two-step pocess, the oxidizing environment and Dsb system of periplasm benefits the formation of disulfide bonds [31]. Further experiments showed that Cel-CD can also be expressed and secreted from *E. coli* K strains, such as *E. coli* BW25113, MG1655, though the level of secretion was much lower. Thus, the application of Cel-CD fusion partner is not strain dependent.

We have proven that the N-terminal sequence of full-length Cel-CD plays a key role in secretion. This indicated that N-terminal 20 amino acid residues of Cel-CD can serve as a carrier for the secretion of heterologous target proteins out of *E. coli*. Fused with Cel-CD or N20, five selected proteins found to secrete into the medium at high levels. The secretion efficiency depends on the properties of the target proteins. Cel-CD appears to be suitable for the secretion of small and low-solubility proteins because of its high solubility and large size in *E. coli*, whereas the N20 peptide is more suitable for large proteins. Notably, all secreted fusion proteins exhibited relative high solubility; fusion proteins with low solubility are not efficiently secreted. We suggest that the solubility of the fusion protein is a prerequisite for secretion, while the N-terminal sequence and three dimensional structure of the protein is a crucial factor. Fusion of heterologous proteins with N20 from Cel-CD can carry the target proteins out of the cell. This is the first report to show that a short peptide can serve as a signal peptide and guide heterologous proteins across both the inner and outer membranes of *E. coli*.

Secretory production of proteins in *E. coli* has many possible applications. For example, a mutagenized protein library could be directly screened on a plate if the protein or its product is directly visible, which can greatly facilitate protein engineering research. Extracellular accumulation of Cel-CD with other cellulosic enzymes may also be useful for the creation of consolidated bioprocess for simultaneous biomass conversion product formation [32]. Finally, secretory production of proteins will simplify the protein production process and will decrease production costs of enzymes and antibodies.

## Conclusions

Recombinant proteins produced in *Escherichia coli* are generally intracellular and often found in the form of inclusion bodies. In this study, a heterologous expressed catalytic domain of a cellulase (Cel-CD) in *E. coli* was found to be secreted out of the cells in large quantities with endo-beta-glycosidase activity. We demonstrated that the N-terminal sequence of Cel-CD played a crucial role in this secretion, which suggests that both Cel-CD and its N-terminus can be employed as carriers for extracellular production of recombinant proteins. Recombinant *E. coli* expressing the fusion protein of Cel-CD and other types of hydrolytic enzymes can be used for creation of consolidated bioprocess.

## Materials and methods

### Bacterial strains and plasmids construction

Strains and plasmids used in this study were shown in Table 1. *E. coli* strain DH5α (Invitrogen) was used for recombinant DNA manipulation. *E. coli* BL21 (DE3) (Novagen) was used as host for the expression of Cel-CD and other recombinant proteins. Cells were cultured in 5 mL Luria Broth (LB) medium for 12 h at 37°C. This seed culture was inoculated to 50 mL LB medium in a 300 mL flask. To induce the expression of the desired proteins, Isopropyl-β-D-thiogalactopyranoside (IPTG) was added to a final concentration of 0.5 mM when OD_600_ reached 0.8. After induction, cells were culture until the indicated time. Antibiotics were added as follows: kanamycin (Kan) 50 μg/mL.

**Table 1:** **The strains and plasmids used in this study**

**Description**		**Source**
**Strains**		
*E. coli* DH5α	F^−^ *endA1 hsdR17(r*_*K*_ *- m*_*K*_ *+) supE44 thi-l λ* ^*−*^ *recA1 gyrA96 ΔlacU169 (Φ80dlacZM15*)	Invitrogen
*E. coli* BL21(DE3)	*hsdS, gal(ΔcIts857 ind1, Sam7, nin5, lacUV5-T7, gene1*	Novagen
**Plasmids**		
pET28a	5.4 kb, f1 ori, T7 promoter, Kan^R^	Novagen
pCel	pET28a, *cel-cd* gene under T7 promoter	This study
pN20	pET28a, 60 bp at 5′ region of *cel-cd* under T7 promoter	This study
pΔ5cel	pET28a, *cel-cd* gene that truncated 15 bp at 5′ region	This study
pΔ10cel	pET28a, *cel-cd* gene that truncated 30 bp at 5′ region	This study
pΔ15cel	pET28a, *cel-cd* gene that truncated 45 bp at 5′ region	This study
pΔ20cel	pET28a, *cel-cd* gene that truncated 60 bp at 5′ region	This study
pΔ30cel	pET28a, *cel-cd* gene that truncated 90 bp at 5′ region	This study
pCelNRN1	pET28a, *cel-cd* gene fusion with *nrn1*from *Homo sapiens*	This study
pCelMBP	pET28a, *cel-cd* gene fusion with *male* from *Escherichia coli*	This study
pCelCBD	pET28a, *cel-cd* gene fusion with *cbd* from *Bacillus circulans*	This study
pCelGlpQ	pET28a, *cel-cd* gene fusion with *glpQ* from *Escherichia coli*	This study
pCelPelC	pET28a, *cel-cd* gene fusion with *pelc* from *Bacillus subtilis*	This study
p20NRN1	pET28a, *n20* gene fusion with *nrn1*	This study
p20MBP	pET28a, *n20* gene fusion with *male*	This study
p20CBD	pET28a, *n20* gene fusion with *cbd*	This study
p20GlpQ	pET28a, *n20* gene fusion with *glpq*	This study
p20PelC	pET28a, *n20* gene fusion with *pelc*	This study
E170Q	pET28a, the mutated *cel-cd* gene under T7 promoter	This study
E170Q&E275Q	pET28a, the mutated *cel-cd* gene under T7 promoter	This study

Cel-CD expression plasmids was created based on pET28a (+) (Novagen) containing the T7 promoter. The DNA sequence encoding the catalytic domain of cellulase (Cel-CD) used in this study was amplified from the genome of *Bacillus sp.* Z-16 (lab collection) by polymerase chain reaction using PrimeSTAR (TaKaRa) with the Cel-CD-*Nco*I-forward and Cel-CD-*Xho*I-Reverse as primers (Table 2). After digestion with *Nco*I and *Xho*I (Fermentas), the amplified DNA fragment was inserted into pET-28a using T4 DNA ligase (NEB), generating plasmid pCel. The DNA sequence encoding N20 amino acid residues of Cel-CD used in this study was amplified from the plasmids pCel by polymerase chain reaction using PrimeSTAR (TaKaRa) with the Cel-CD-*Nco*I-forward and Cel-20-*Bam*HI-Reverse as primers.

**Table 2:** **The primers used in this study**

**Primers**	**Sequence**
Cel-CD-*Nco*I-Forward	5′-TTTT*CCATGG*AAGGAAACACTCGTGAAGA-3′
Cel-CD-*Xho*I-Reverse	5′-TTTT*CTCGAG*AAGTACTTTCGTGTATTTTG-3′
Cel-CD-*Bam*HI-Reverse	5′-TTTT*GGATCC*AAGTACTTTCGTGTATTTTG-3′
Cel-20-*Bam*HI-Reverse	5′-TTTT*GGATCC*GCGTTTAACATTGTCATTAC-3′
Cel-CD5-*Nco*I-Forward	5′-TTTT*CCATGG*AAGACAATTTTAAACATTT-3′
Cel-CD10-*Nco*I-Forward	5′-TTTT*CCATGG*AACATTTATTAGGTAATGACAA-3′
Cel-CD15-*Nco*I-Forward	5′-TTTT*CCATGG*ACAATGTTAAACGCCCTTC-3′
Cel-CD20-*Nco*I-Forward	5′-TTTT*CCATGG*AACCTTCTGAGGCTGGCGCATT-3′
Cel-CD30-*Nco*I-Forward	5′-TTTT*CCATGG*AAGTCGATGGACAAATGACA-3′
NRN1-*Bam*HI-Forward	5′-TTTT*GGATCC*GCTGGTAAATGTGACGCCG-3′
NRN1-*Xho*I-Reverse	5′-TTTT*CTCGAG*TTAACCATTGCCCGAGCCG-3′
MBP-*Bam*HI-Forward	5′-TTTT*GGATCC*ATCGAAGAAGGTAAACTGG-3′
MBP-*Xho*I-Reverse	5′-TTTT*CTCGAG*TTACTTGGTGATACGAGTC-3′
GlpQ–*Bam*HI-Forward	5′-TTTT*GGATCC*GCGGACAGCAACGAAAAAA-3′
GlpQ-*Xho*I-Reverse	5′-TTTT*CTCGAG*TTACTCTTTATTAAGAAATT-3′
CBD–*Bam*HI-Forward	5′-TTTT*GGATCC*GACCAGGTCAGCGTCCGCTT-3′
CBD-*Xho*I- Reverse	5′-TTTT*CTCGAG*TTATGGCTGCCAATTCACGT-3′
pelC–*Bam*HI-Forward	5′-TTTT*GGATCC*GCAGATAAGGTTGTCCACGA-3′
pelC-*Xho*I- Reverse	5′-TTTT*CTCGAG*TTAGAACTGGGTGTTATTAT-3′
Glu160Gln-Forward	5′-TTATTTATGAGTTAGCGAAT*CAG*CCAAGTAGTA-3′
Glu160Gln-Reverse	5′- GATTCGCTAACTCATAAATAATGTGTGGATTGT-3′
Glu274Gln-Forward	5′-GAGTAGCGGTATTTGCGACA*CAA*TGGGGAACGA-3′
Glu274Gln-Reverse	5′-GTGTCGCAAATACCGCTACTCCGTTTTCTAACG-3′

The DNA sequence encoding Cel-CD with 5 amino acid residues deletion at the N-terminus was amplified from the plasmids pCel by polymerase chain reaction using PrimeSTAR (TaKaRa) with the Cel-CD5-*Nco*I-Forward and Cel-CD-*Xho*I-Reverse as primers, generating plasmids p*Δ*5cel. The same method was used to construct p*Δ*10cel, p*Δ*15cel, p*Δ*20cel and p*Δ*30cel.

To fuse the target protein genes to the 3′ end of *cel-cd*, the DNA fragments were amplified by PCR, and were digested with restriction enzyme *Bam*HI and *XhoI* on the both ends*.* The resulting fragments were then ligated to the vector pET-28a/*cel* by T4 DNA ligase to obtain the objective plasmids. The same method was used to construct N20 fusion proteins.

### Cel-CD activity assay

The hydrolytic activity of the secreted Cel-CD was determined by Dinitrosalicylic Acid (DNS) method. 0.1 mL culture medium was added to 2 mL Britton-Robinson buffer containing 1% CMC-Na in a test tube. Reaction mixture was incubated at 37°C for 30 min, then was added with 2 mL DNS reagent and boiled for 5 min before it was diluted to 12.5 mL. The enzyme activity was measured with a spectrophotometer at 540 nm.

### Protein quantification

The total secretory proteins were quantified by Bradford method using Easy Protein Quantitative kit (Lot #G10322, TransGen Biotech). The proteins strained with Coomassie were detected by the UV spectrophotometer at 595 nm. The protein samples were loaded on 12% or 15% SDS-PAGE and stained with Coomassie dye. The stained gels were scanned by Scanner and the target protein bands were normalized and quantified by Bio-Rad Quantity One Version 4.0.

### SDS-PAGE and western blotting

Cells were harvested at indicated time after induction and washed once with 1 mL of 100 mM MOPS (pH 7.0). The secreted protein samples were obtained directly by centrifugation. The cell pellets were then disrupted by ultrasonification and centrifuged to obtain the total soluble protein samples. The samples were then prepared by adding an equal volume of electrophoresis loading buffer. Proteins were separated on 12% SDS-PAGE and either stained with Coomassie dye or transferred to a PVDF membrane (100 V, 1 h) for immunoblotting, blocked with 5% (W/V) BSA in PBS/Tween, and probed using the appropriate antibody. Antibody to Cel-CD (Sigma, 1:2,000) was used for the detection of secreted Cel-CD in the culture medium and cells. Antibody to MalE (Sigma, 1:5,000) and GroEL (Sigma, 1:10,000) were used for the control of potential lysis or leakage from the periplasmic space (MalE) and cytoplasmic (GroEL).

### Site-directed mutagenesis of Cel-CD

Site-directed mutagenesis mutants were constructed using Easy Mutagenesis System Kit (TransGene). The primers Glu160Gln-Forward/Reverse were included 5′end overlap region, mutation site and 3′ end extension region. In a certain system, the target DNA vectors containing the *cel-cd* gene were amplified by PCR and the templates were digested by DMT enzyme. After purified by gel extraction, the target vectors containing the E160Q mutant and E160Q&E274Q mutant was obtained. The Gln in the active sites of Cel-CD (Gln160 and Gln274) were all mutated to Glu. All the resulting constructs were confirmed by DNA sequencing and hydrolytic activity determination.

### Transmission electron microscopy (TEM)

*E. coli* cells containing the target plasmids were collected by centrifuging at 4,000 g for 10 min. Pelleted cells were fixed by 2.5% glutaraldehyde and 1% osmic acid. After gradually dehydrated by ethyl alcohol, the cells were embedded in Epon-812 araldite. The samples were cut into 70 nm thick slice, stained using 2% uranyl acetate and lead citrate, and observed by the transmission electron microscope at 200 kV. The resulting microscopic images were photographed.

### Analysis of the N-terminal sequence

The protein samples were purified and separated by 2D-SDS PAGE, and were transferred to polyvinylidene difluoride (PVDF) blotting membrane. The N-terminal sequence of the protein was analyzed by EDMAN degradation method [33] using the PROCISE491 protein sequencer by the Center for Protein Science, Peking University.

### Congo red staining

To determine the hydrolytic activity of Cel-CD, an agarose plate-based assay was used. 1% (wt/vol) Carboxymethylcellulose (CMC-Na) was added in the agarose plate as a substrate (0.8%, wt/vol). Samples containing the Cel-CD were loaded into the hole of plate. The plate was incubated at 30°C for 3 h, then flooded with 0.1% (wt/vol) Congo red solution and stained for 15 min at room temperature. The dye was removed, and 5 mL of water was used to wash the plate. Finally, 5 mL 0.9% NaCl solution was applied for 15 min, and the plates were then dried and photographed.

### Subcellular fractionation

Periplasmic protein was separated from cytoplasm by the osmotic shock method [34]. The cells were harvested by centrifugation (6,000 g for 5 min) and were suspended in 100 mM Tris–HCl containing 20% sucrose and 1 mM EDTA (pH 8.0), then pelleted by centrifugation (8,000 g for 5 min) followed by re-suspension in ice-cold water for 10 min. After the addition of MgCl_2_ to a final concentration of 1 mM, the cell suspension was incubated on ice for a further 10 min before being pelleted by centrifugation (8,000 g for 5 min).
